# The Assessment and Relationship Between Quality of Life and Physical Activity Levels in Greek Breast Cancer Female Patients under Chemotherapy

**DOI:** 10.3390/sports8030032

**Published:** 2020-03-11

**Authors:** Maria Maridaki, Argyro Papadopetraki, Helen Karagianni, Michael Koutsilieris, Anastassios Philippou

**Affiliations:** 1 Faculty of Physical Education and Sport Science, National and Kapodistrian University of Athens, 17237 Dafne, Greece; 2 Medical School, National and Kapodistrian University of Athens, 11527 Athens, Greece; argpapa@med.uoa.gr (A.P.); elenikara13@gmail.com (H.K.); mkoutsil@med.uoa.gr (M.K.); tfilipou@med.uoa.gr (A.P.)

**Keywords:** breast cancer, chemotherapy, physical activity, quality of life, exercise, QoL, treatment

## Abstract

A growing body of evidence suggests that physical activity (PA) can be a complementary intervention during breast cancer (BCa) treatment, contributing to the alleviation of the chemotherapy-related side-effects. The purpose of this study was to assess physical activity (PA) levels and quality of life (QoL) parameters of BCa patients undergoing chemotherapy and compare them with healthy controls. A total of 94 BCa female patients and 65 healthy women were recruited and self-reported QoL and PA levels. The results reveal that women suffering from BCa spent only 134 ± 469 metabolic equivalents (MET)/week in vigorous PAs compared with the healthy females who spent 985±1508 MET/week. Also, BCa patients were spending 4.62±2.58 h/day sitting, contrary to the 2.34±1.05 h/day of the controls. QoL was scored as 63.43±20.63 and 70.14±19.49 while physical functioning (PF) as 71.48±23.35 and 84.46±15.48 by BCa patients and healthy participants, respectively. Negative correlations were found between QoL and fatigue, PF and pain, and fatigue and dyspnea, while a positive correlation was found between QoL and PF. This study indicated that the BCa group accumulated many hours seated and refrained from vigorous Pas, preferring PAs of moderate intensity. Additionally, BCa patients’ levels of functioning and QoL were moderate to high; however, they were compromised by pain, dyspnea and fatigue.

## 1. Introduction

According to the World Health Organization (WHO), cancer is a leading cause of mortality worldwide, while approximately one out of six deaths is due to cancer. In both sexes, lung cancer is the most commonly diagnosed malignancy and the most frequent cancer leading to death. On the other hand, among females, breast cancer (BCa) constitutes the most commonly diagnosed cancer, as well as the first in mortality rate [1]. Epidemiological studies revealed that in spite of the fact that BCa accounts for about 30% of all cancer diagnoses in women [2], the overall 5-year survival rate is over 90% for survivors diagnosed with BCa stage I or II [3]. 

The increased survival rates due to advancements in cancer detection and medical care indicate that cancer should be handled as a chronic disease that requires long term management to maintain patients’ quality of life [4]. It is well established that standard medical care for BCa, including surgery, chemotherapy, radiotherapy and hormonal therapy, is associated with adverse effects on cardiorespiratory, musculoskeletal, nervous and endocrine physiological systems [5,6,7]. In particular, cardiotoxicity, cancer-related fatigue, muscle atrophy, cachexia, peripheral neuropathy, immune system dysfunction and altered body composition are some of the reported complications that result in a diminished quality of life (QoL) of patients, while interfering with their ability to carry out regular daily living activities [8,9,10,11]. Moreover, general pain and fatigue belong to the most frequently experienced symptoms that cancer patients undergoing treatment exhibit and these symptoms are related to the severity of the disease [12].

However, an increasing body of evidence suggests that prescribed exercise during and after cancer treatment may attenuate many of these adverse effects and mitigate several symptoms, constituting a safe complementary therapeutic intervention for cancer patients [12]. In addition to the studies that suggest the preventive role of physical activity against BCa risk [13,14], there is also evidence supporting that regular exercise also reduces the risk of disease recurrence for several types of solid tumors including BCa. These inhibitory effects of regular exercise are probably mediated by different mechanisms that alter the tumor microenvironment [15,16]. 

The American College of Sports Medicine (ACSM) and the American Cancer Society (ACS) recommend that BCa patients should avoid remaining inactive and aim to return to their normal daily routine as soon as possible after diagnosis and during the treatment of the disease. For instance, BCa patients should be encouraged to accumulate at least 150 or 75 min of moderate or vigorous aerobic exercise per week, respectively, and include resistant training exercises two to three times per week [17]. The compliance to these guidelines is really important for the individuals subjected to cancer treatment, because, as in a chronic disease, so in cancer, there is a dose–response relationship between physical activity (PA) levels and health benefits gained [18].

Despite the abovementioned recommendations, current research evidence suggests that the majority of people living with cancer do not participate in PAs and they adopt sedentary behavior [19,20]. The purpose of the present study was to assess QoL and PA levels of BCa female patients living in Greece and undergoing chemotherapy, and to compare them with healthy age- and sex-matched controls. 

## 2. Materials and Methods

### 2.1. Ethical Approval

All volunteers provided written informed consent to participate in this cross-sectional observational study, which was approved by the seven-member Ethics Committee of the Medical School of the National and Kapodistrian University of Athens. All data were collected and handled according to privacy law regulations.

### 2.2. Subjects

A total of 159 females, aged from 42 to 71 years, voluntarily participated in the study. From them, 94 women (age: 57.25 ± 13.59 years) were newly diagnosed with breast cancer for first time, stage I-III, and had already started to receive first-line chemotherapy, while 65 healthy women (age: 49.60 ± 7.80 years) served as a control group. The patients were recruited in close collaboration with the attendant physicians from three different Greek hospitals, from the October of 2017 to the October of 2018, and filled the questionnaires during their first regimen of chemotherapy while no exclusion criteria were set according to the type of surgery that had preceded. The women who comprised the control group were recruited in the same chronological period and they should have never been diagnosed with cancer. Moreover, all participants should speak and read Greek. 

### 2.3. Data Collection 

All participants filled in the structured questionnaires, while their body height and body mass were measured in order for their body mass index (BMI) to be calculated. Participants were instructed to answer all the questions as carefully and honestly as possible, while an investigator was available for providing clarifications for any possible questions raised regarding the way that the questionnaires should be filled in. 

#### 2.3.1. Somatometric Characteristics

Body height was measured with the subject standing in bare feet with her back towards a height measuring rod and body mass was measured with an electronic precision balance with two decimals. Body mass index (BMI) was then calculated according to the following formula: BMI = body mass (kg) / body height ^2 (m^2^). Individuals were considered to be of normal body weight if their BMI was between 20 and 24.9, while they were considered as underweight if their BMI was lower than 20. If BMI was in the range between 25 and 29.9, or higher than 30, the individual was considered as overweight or obese, respectively [21].

#### 2.3.2. Quality of Life

Quality of life was self-estimated by the BCa patients and the healthy controls, using the EORTQ-QLQ-C30 or the SF-36 Health Survey Version 3.0 questionnaire, respectively [22,23,24]. Specifically, EORTQ-QLQ-C30 is a cancer-specific questionnaire that incorporates global health status/QoL scale, common symptom scales and physical, emotional, cognitive, role and social functioning scales. For example, some of the items the questionnaire focuses on are pain, fatigue, sleep, concentration, appetite etc. In this particular questionnaire, the score in each scale ranges from 0 to 100. The higher the score on the functional scales or the global health status is, the greater the level of functioning and QoL. Reversely, a high score in the symptom scale reflects a high level of symptomatology. 

Similarly, SF-36 is a 36-item questionnaire that covers eight health domains: physical functioning, pain, fatigue, role limitations due to physical health problems, role limitations due to emotional problems, emotional well-being, social functioning and general health perceptions. Each item of this questionnaire is also scored on a 0 to 100 scale and in all scales a higher score defines a more favorable health status. For instance, a higher score in the fatigue scale actually represents less fatigue. The two questionnaires, SF-36 and EORTQ-QLQ-C30, have the same way of scoring and interpreting the results in the scales general QoL, Physical Functioning, Emotional Functioning, Social Functioning and Role Functioning. Thus, the comparisons of QoL were based on the similarity in scales (0–100, with a higher score indicating better health) and not on actual survey questions or summary calculations.

#### 2.3.3. Exercise Behavior

Current PA levels were self-reported by the participants using the short version of the International Physical Activity Questionnaire (IPAQ). IPAQ assesses the duration and the intensity of PAs as well as the time spent sitting in daily lives, while it is considered to estimate the total weekly energy expenditure in MET-min per week. Activities that require up to 3 METs have been defined as light-intensity PAs, activities that range from 3 to 6 METs have been categorized as moderate-intensity PAs, whereas those that require more than 6 METs were defined as vigorous-intensity PAs [25].

### 2.4. Statistical Analysis

Statistical analysis was conducted using Graphpad Prism Version 5.03 (GraphPad Software, Inc., San Diego, CA, USA). For all quantitative variables, descriptive analysis was employed by mean and standard deviation (MEAN ± SD), while evaluation of the potential differences between the two independent groups (i.e., BCa vs Control group) was performed with a two-tailed, unpaired Student T-test. Pearson parametric correlation coefficient was utilized to determine any potential associations between the continuous variables—physical activity and QoL. The level of statistical significance was set at P < 0.05.

## 3. Results

### 3.1. Somatometric Characteristics

The somatometric characteristics of the participants in each group (i.e., BCa patients and healthy controls) are shown in Table 1. Height was 1.61 ± 0.05 m and 1.65 ± 0.04 m in the BCa and control group, respectively, while body mass was 69.49 ± 12.67 kg in BCa patients and 69.04 ± 5.25 kg in healthy controls. BMI was used for the classification of participants as underweight, normoweight, overweight or obese. These results reveal that BCa patients’ BMI was 26.63 ± 5.27 kg/m^2^, categorizing them as overweight, by contrast with the healthy females in the control group whose BMI was marginally normal (25.30 ± 3.95 kg/m^2^). 

### 3.2. Quality of Life

#### 3.2.1. Control Group

**Figure 1:**
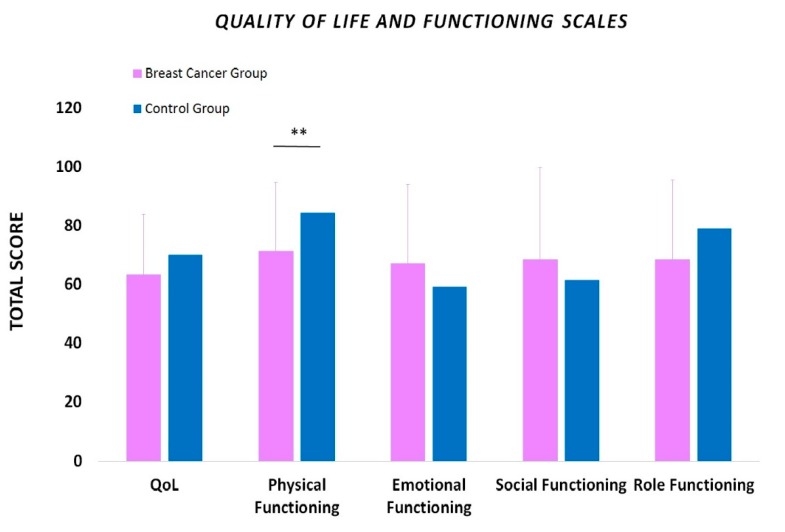
Self-estimation of the overall quality of life (QoL) and its functional parameters in women undergoing chemotherapy for breast cancer compared with healthy controls. Data are presented as mean ± SD. **: Significantly different at p < 0.01.

**Figure 2:**
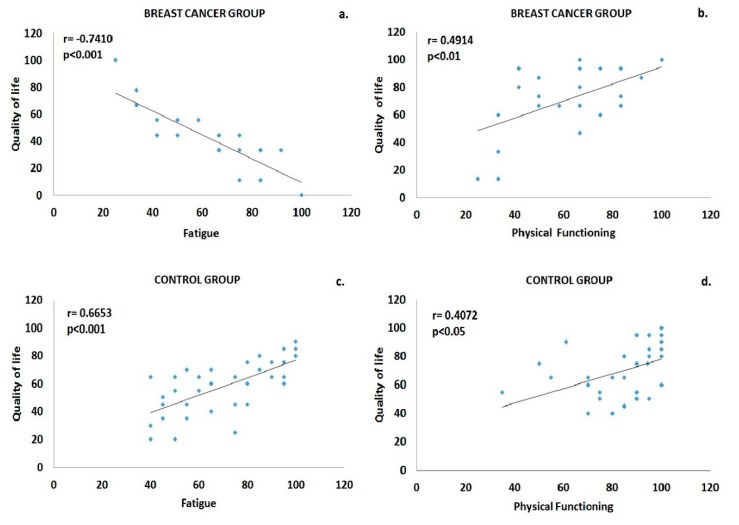
Correlational analyses revealed significant associations, among others (see text for details), between fatigue and quality of life (**a**,**c**), as well as between physical functioning and quality of life (**b**,**d**), both in the breast cancer and the control group.

Healthy females who served as the control group self-evaluated their QoL using the SF-36 Health Survey Version 3.0. Regarding their general QoL, the participants scored 70.14 ± 19.49, while for their physical, emotional, social and role functioning their score was 84.46 ± 15.48, 59.33 ± 17.83, 61.79 ± 27.05 and 79.17 ± 29.76, respectively (Figure 1). Moreover, in the symptom scales, pain was scored with 70.42 ± 22.93 and fatigue with 58.06 ± 12.23. Positive correlations were revealed between physical functioning and pain (r = 0.4432, p = 0.007), fatigue (r = 0.4847, p = 0.003), emotional functioning (r = 0.4133, p = 0.012) and role functioning (r = 0.3869, p = 0.020). Positive correlations were also found between QoL and the scales of physical functioning (r = 0.4072, p = 0.014) and fatigue (r = 0.6653, p = 0.00001), (Figure 2).

#### 3.2.2. Breast Cancer Group

Similarly to the control group, the women of the BCa group self-estimated their QoL using the EORTC-QLQ-C30 Questionnaire. Women in the BCa group scored their physical functioning significantly lower compared with the healthy controls (71.48 ± 23.35 vs 84.46 ± 15.48; p<0.01). However, their overall QoL, as well as their emotional, social and role functioning score, was 63.43 ± 20.63, 67.13 ± 27.02, 68.52 ± 31.31 and 68.98 ± 26.77, respectively, revealing no significant differences with the control group (p > 0.05) (Figure 1). It is noted that comparisons between the BCa and control group were performed only between the above-mentioned scales, since the rest of them in each questionnaire have a different way of scoring.

As far the symptomatology is concerned, fatigue was scored at 42.28 ± 20.54, dyspnea at 25.93 ± 28.85 and pain at 19.44 ± 24.40. A negative correlation was found between QoL and fatigue (r = −0.7410, p = 0.00001), as well as between physical functioning and pain (r = −0.6149, p = 0.0001), fatigue (r = −0.6661, p = 0.0001) and dyspnea (r = −0.3320, p = 0.0493), (Figure 2). In contrast, a positive correlation was revealed between physical functioning and QoL (r = 0.4914, p = 0.0024), social functioning (r = 0.5954, p = 0.0001) and emotional functioning (r = 0.3663, p = 0.0263) (Figure 2). 

### 3.3. Exercise Behavior

**Figure 3:**
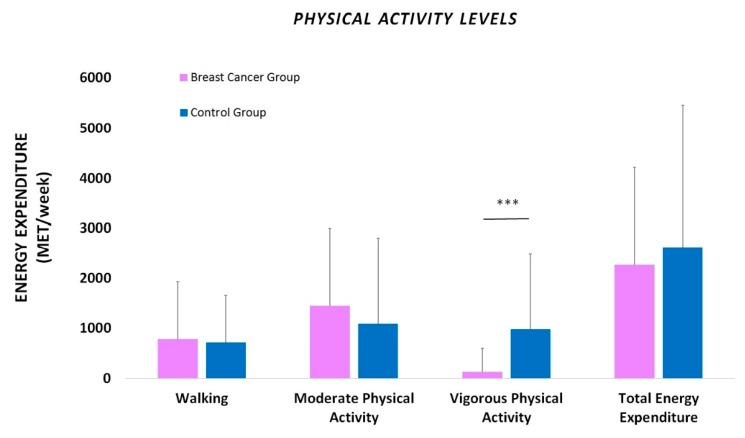
Self-reported physical activity levels (International Physical Activity Questionnaire (IPAQ)) in women undergoing chemotherapy for breast cancer compared with healthy controls, expressed in MET-min per week. Data are presented as mean ± SD.***: Significantly different at p<0.001.

Exercise behavior was self-reported by all participants using the International Physical Activity Questionnaire (IPAQ) (Figure 3). Specifically, BCa patients exhibited a total energy expenditure of 2267 ± 1965 MET-min/week, while healthy controls spent 2630 ± 2840 MET-min/week, showing no significant differences between groups (p > 0.05). In particular, no significant differences (p > 0.05) were found between the two groups in the time spent walking (BCa group: 782 ± 1,153 MET-min/week vs Control group: 721 ± 950 MET-min/week). A similar (p > 0.05) energy expenditure was also spent in moderate PAs by both BCa and control group, i.e., 1460 ± 1549 vs 1089 ± 1724 MET-min/week, respectively. Interestingly, on the other hand, BCa patients were found to participate in vigorous PAs disproportionally less than the control group, expending only 134 ± 469 MET-min/week, as opposed to the control group that spent 985 ± 1,508 MET-min/week in high-intensity activities (p < 0.001). It is noted that moderate PAs require intermediate physical effort and make breathing somewhat harder than normal, while vigorous PAs need excess physical effort, increasing breath rate. 

Furthermore, BCa patients were found to spend more time sitting during the day (4.20 ± 2.76 h/day) in comparison with the control group (3.16 ± 1.25 h/day), (p < 0.05). Again, it is noted that sedentary time includes time spent sitting or lying down during work and leisure, or at home and excludes sleeping hours. 

### 3.4. Associations between Exercise Behavior and Quality of Life

**Figure 4:**
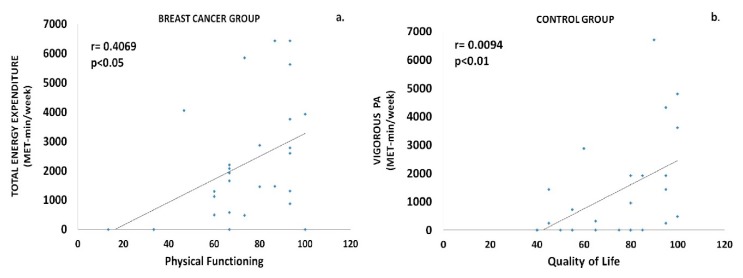
Correlational analyses showed significant associations, among others (see text for details), between (**a**) total energy expenditure and physical functioning in the breast cancer group as well as between (**b**) the engagement in vigorous PAs and the quality of life, in the control group.

In the BCa group, a positive correlation was demonstrated between physical functioning and total energy expenditure (r = 0.4069, p = 0.0316) (Figure 4a), as well as between QoL and participation in vigorous PAs (r = 0.3985, p = 0.0357). Similarly, a positive correlation was also found in the control group between the engagement in vigorous PAs and QoL (r = 0.4993, p = 0.0094) (Figure 4b), as well as between vigorous PAs and physical functioning (r = 0.5149, p = 0.0071).

## 4. Discussion

The aim of the present study was to identify the levels of PA and the perceived QoL, investigating their potential interactions, in females undergoing chemotherapy due to BCa diagnosis, and to compare them with healthy females of the same age. 

Our main findings demonstrate that women suffering from BCa and undergoing chemotherapy were willing to exercise and they participated in regular PAs, exhibiting weekly energy expenditure levels similar to those of sex- and aged-matched healthy individuals. However, they preferred to exercise in low or moderate intensities, showing significantly lower levels of MET-min per week expended in high intensity PAs compared with the healthy controls. These findings are in agreement with previous studies implying that cancer patients demonstrate lower levels of vigorous-intensity PAs post than before diagnosis [26,27]. Even though it has been established that high-intensity activities can safely be performed by cancer patients, offering different health benefits than those derived from the conventional exercise programs, cancer patients appear to hesitate to participate in vigorous PAs [28]. On the other hand, cancer-related fatigue and general pain probably exacerbate the overall burden of the disease and the therapeutic interventions, making participation in more intense physical activities difficult, especially for those patients with more advanced stages of the disease. 

Moreover, our study showed that although the BCa patients were exercising in general, they accumulated many hours per day sitting down, not only at work but also at home, since many patients often interrupted their work during chemotherapy sessions, thus spending more hours per day seated at home, which may result in their overweight phenotype. These findings strengthen the evidence from previous studies which supported the hypothesis that an increased BMI is associated with a sedentary lifestyle after cancer diagnosis [29]. Since an increased body weight has been associated with a higher risk of disease recurrence and reduced survival, all cancer patients should not only avoid remaining physically inactive but also they need to follow the specific exercise recommendations, so as to optimize their health exercise benefits [30]. 

Regarding the QoL, our study showed that BCa patients exhibited moderate levels of perceived QoL, similarly to the control group. More specifically, a strong negative correlation was found between QoL and fatigue as expected, highlighting the fact that cancer-related fatigue remains a huge barrier to patients’ daily life [31,32]. In addition, negative correlations were also found between physical functioning and the side effects of the disease, such as pain, fatigue and dyspnea, indicating that these symptoms compromise patients’ functional capacity and QoL [33]. Similar associations between the symptomatology and functional scales were also observed in the control group, indicating that the above-mentioned clinical symptoms influence the individuals’ daily life independently of the disease.

Moreover, with regard to the relationship between exercise behavior and QoL, a positive correlation was found between participation in vigorous PAs and QoL, as well as between total energy expenditure and physical functioning in women with BCa. These findings corroborate a large body of evidence supporting the hypothesis that a greater energy expenditure during the week leads to a better functional ability, while participation in more intense activities implies a better self-evaluated QoL [34,35]. Moreover, the strong positive correlations found between physical, social and emotional functioning further support previous findings that mental health symptoms and isolation are followed by a poor functional ability in cancer patients [36,37].

Putting all the above findings together, it appears that new approaches are urgently needed to improve tolerance and reduce the adverse effects of chemotherapy in cancer patients [38]. Physical activity interventions should be incorporated in cancer non-pharmaceutical treatments during chemotherapy, since the worst side effects of cancer therapy are experienced during this period, while exercise can mitigate unfavorable changes in various physiological systems and their consequent symptoms [39,40,41]. Clinical physicians are proposed to assess, advise and refer cancer patients to exercise [19,42,43]. 

## 5. Conclusions and Future Perspectives

The outcomes of the present study unveil a close relationship between exercise behavior and QoL in breast cancer patients; however, there remain challenging issues to be further addressed. Future research lines of investigation should focus on the dose-dependent effects of physical activity and on revealing the optimum dose as well as the potential maximum and minimum thresholds of the cancer patients for benefit from physical activity. Furthermore, it remains a challenge to elucidate whether cancer type, timing of physical activity and its specific components influence the effectiveness of exercise and its interactions with cancer outcomes. For instance, in order for vigorous physical activities to be realistically adopted and sustained by those patients during their treatment, a mode of short-duration high-intensity physical exercise with adequate breaks might be a more applicable suggestion for them, so as to take advantage of the time-effective, beneficial effects of vigorous activities on their quality of life and physical functioning. Since physical activity is an important factor for cancer prevention and treatment, policy makers, public health professionals, health care providers, and exercise scientists should all communicate and promote the benefits of physical activity for both cancer prevention and control, and work together with other stakeholders to improve the health and quality of life of cancer patients.

## Figures and Tables

**Table 1:** Somatometric characteristics of breast cancer patients and healthy participants (control group).

Participants’ Characteristics	Breast Cancer Group (n=94)	Control Group (n=65)
Age (yrs)	57.25 ± 13.59	49.60 ± 7.80
Body Mass (kg)	69.49 ± 12.67	69.04 ± 5.25
Body Height (m)	1.61 ± 0.05	1.65 ± 0.04
Body Mass Index (kg/m^2^)	26.63 ± 5.27	25.30 ± 3.95

Data are presented as mean ± SD. No statistically significant differences were found between groups (p>0.05).
